# Expression, characterization, and application of human-like recombinant gelatin

**DOI:** 10.1186/s40643-024-00785-1

**Published:** 2024-07-17

**Authors:** Xiaoping Song, Tao Chu, Wanru Shi, Jingyan He

**Affiliations:** 1 Department of Pharmacy, Anhui Medical College, Hefei, Anhui 230061 China; 2 Anhui Engineering Research Center of Recombinant Protein Pharmaceutical Biotechnology, Hefei, Anhui 230022 China

**Keywords:** Recombinant human gelatin, Monomer protein sequence, *Pichia pastoris*, Characterization, Three- dimensional culture, Biomedical materials

## Abstract

Gelatin is a product obtained through partial hydrolysis and thermal denaturation of collagen, belonging to natural biopeptides. With irreplaceable biological functions in the field of biomedical science and tissue engineering, it has been widely applied. The amino acid sequence of recombinant human-like gelatin was constructed through a newly designed hexamer composed of six protein monomer sequences in series, with the minimum repeating unit being the characteristic Gly-X-Y sequence found in type III human collagen α1 chain. The nucleotide sequence was subsequently inserted into the genome of *Pichia pastoris* to enable soluble secretion expression of recombinant gelatin. At the shake flask fermentation level, the yield of recombinant gelatin is up to 0.057 g/L, and its purity can rise up to 95% through affinity purification. It was confirmed in the molecular weight determination and amino acid analysis that the amino acid composition of the obtained recombinant gelatin is identical to that of the theoretically designed. Furthermore, scanning electron microscopy revealed that the freeze-dried recombinant gelatin hydrogel exhibited a porous structure. After culturing cells continuously within these gelatin microspheres for two days followed by fluorescence staining and observation through confocal laser scanning microscopy, it was observed that cells clustered together within the gelatin matrix, exhibiting three-dimensional growth characteristics while maintaining good viability. This research presents promising prospects for developing recombinant gelatin as a biomedical material.

**Supplementary Information:**

The online version contains supplementary material available at 10.1186/s40643-024-00785-1.

## Introduction

Natural gelatin is a collagen degradation product extracted from animal skin, bones, and connective tissues. It enjoys advantages such as low immunogenicity, good biodegradability, and biocompatibility (Wei et al. 2021). Gelatin microspheres (GMs) prepared from this material are spherical particles in the micro-nano scale that can uniformly disperse drugs or other components within the gelatin matrix. Currently, they are widely used in tissue engineering for various applications including tissue regeneration, drug delivery systems, and cell delivery scaffolds (Sabzi et al. 2020; Yang et al. 2021; Zhang et al. 2021). Structurally, gelatin contains an RGD amino acid sequence (Arg-Gly-Asp) that promotes cell adhesion (You et al. 2020). Additionally, its side chains contain numerous functional groups such as hydroxyl groups, carboxyl groups, and amino groups which allow for structural modifications through covalent or non-covalent grafting of other active moieties to achieve functionalization(Won and Kim 2009). Furthermore, gelatin microspheres features a porous structure that facilitates nutrient transfer and waste removal due to their large surface area available for cell attachment(Gu et al. 2023; Wu et al. 2022). Their injectability enables them to be implanted into irregular bone defect areas of patients (Cai et al. 2023; Yang et al. 2022), with the capability of carrying cells or bioactive factors to accelerate bone tissue repair and regeneration (Kudva et al. 2019; Yang et al. 2022). However, despite the excellent properties of natural gelatin mentioned above, limitations still exist, such as uneven pore distribution and poor shear mechanical performance(Gao et al. 2022). Due to its potential as a carrier of pathogenic microorganisms, natural gelatin cannot be widely used as an injectable drug carrier in clinical and tissue engineering applications, which poses certain limitations and challenges in these fields (Cherim and Srbu 2020). Therefore, only by properly designing the structure of gelatin and providing appropriate spatial organization with excellent biocompatibility can we overcome the current obstacles that hinder its extensive use in clinical and tissue engineering(Ma et al. 2022; Myllyharju et al. 2000; Olsen et al. 2001).

The afore-mentioned issues can be partly addressed with the use of genetic recombination technology to synthesize recombinant gelatin (Xiang et al. 2021). Currently, there is limited literature on the synthesis of recombinant gelatin, with most references relying on biosynthesis methods for producing recombinant collagen. In the employment of genetic recombination technology, it is inevitable to select an appropriate protein expression system and design target gene fragments. It is a promising choice to utilize expression systems such as animal and plant cells, yeast, and *Escherichia coli* for expressing collagen proteins and combining them with high-density fermentation techniques for large-scale production. Zhinan et al. (2016) used *E. coli* as a host to conduct small-scale production of human-like collagen in a 10 L bioreactor induced by 0.1 mM isopropyl-β-D-thiogalactoside (IPTG) under conditions of 28 °C, resulting in a yield of 0.26 g/L. Although *E. coli* is the most common prokaryotic host, its expression system generally has three major drawbacks: potential endotoxin generation making it difficult to apply expressed products clinically; target proteins often expressed in inclusion forms, making purification challenging; and its incomplete post-translational modification system, thereby reducing the biological activity of expressed products among other aspects. In order to achieve better performance of recombinant collagen and gelatin products, it is necessary to ensure that they undergo post-translational modifications (such as glycosylation) by engineered strain, which can only be achieved in eukaryotic expression systems. Therefore, gene engineering technology should be used to construct a recombinant yeast secretion system for the expression of human collagen and gelatin, so as to achieve superior performance and broader application prospects. Many breakthroughs have been made in related research at home and abroad (Liu et al. 2012; Wang et al. 2014). Olsen et al. used *Pichia pastoris* to express a small molecular weight recombinant human-derived gelatin containing only 101 amino acids, which was fermented after high-copy screening with a yield of up to 1.47 g/L(Olsen et al. 2005). After purification, this gelatin can be used as an alternative to animal-derived collagen as a vaccine stabilizer. Liu et al. optimized the expression of a recombinant human-derived collagen containing 599 amino acids using the *P. pastoris* expression system in a 12.5 L fermentation tank, achieving an expression level of 14.49 g/L(Liu et al. 2012). Adopting *P. pastoris* as the host combined with promoter engineering and high-density fermentation technology, Xiang (2022) achieved soluble secretion expression of recombinant type III-like human collagen through two-stage feeding method in a 5 L bioreactor with the highest yield reaching 1.05 g/L(Xiang et al. 2022) .

Another noteworthy issue in the application of gene recombination technology is the design of gene fragments for recombinant collagen. There are three options available: the complete sequence of human collagen, partial sequences of human collagen, and gene fragments similar to human collagen. Such challenges as low expression levels and difficulties in secretion may arise in the use of genetic recombination technology to express the complete human collagen protein. (He et al. 2014; Xiang et al. 2021). Nevertheless, these limitations can be overcome through small molecular weight fragments or active segments to express human collagen proteins, which should become a new strategy (Ma et al. 2022; Olsen et al. 2005).

The molecular composition of type III collagen is homotrimer, with the (Gly-X-Y) n tripeptide repeat sequence being the main feature of the helical region (Wang et al. 2022). Additionally, recombinant human type III collagen has practical applications in promoting extracellular matrix (ECM) remodeling and upregulating the synthesis of type I and III collagens in vivo (Wang et al. 2022). However, natural type III collagen is difficult to express due to its large molecular weight and insolubility. For proteins that are difficult to express, strategies such as expressing small molecular weight fragments or active fragments, and utilizing soluble tags for fusion expression can be used to increase yields.

For this study, an active segment based on human type III collagen was designed as a monomer and six monomers were introduced into an expression vector to construct a genetically engineered strain capable of expressing recombinant gelatin. High-purity recombinant gelatin was successfully obtained through fermentation and purification processes. The characterization and structural studies of this recombinant gelatin lay the foundation for its use as a biomedical material scaffold for 3D cell culture.

## Materials and methods

### Strains and plasmids

The strains and vectors used in our work are shown in Table 1. *E. coli* TOP10 and GS115 strains were utilized as cloning and expression hosts, respectively. The GS115 and pPICZα-B were stored in our laboratory.

**Table 1:** Strains and plasmids used in this study

Strains/ Plasmids	Relevant characteristics	Applications	Sources
Strains E. coli TOP10	F-mcrAΔ(mrr-hsdRMS-mcrBC) φ80 lacZΔM15 ΔlacX74 recA1 araΔ139 Δ(ara-leu)7697 galUgalKrpsL (StrR) endA1 nupG.	Gene cloning host	General Biology
*P. pastoris* GS115	host strain	host strain	Lab stock
*P. pastoris* GS115(*gel*6)	The target gene (name *gel*6) was integrated into Aox1 site of GS115.	Gene expression host	This study
**Plasmids** pUC57‑*gel6*	The pUC57 vector carrying target gene *gel*6, Amp	Gene clonal vector	Synthesized by Universal Biotech Co., Ltd
pPICZα-B	Contains AOX1 promoter for tightly regulated, methanol-induced expression of the gene, Zeocin^R^	Gene expression vector	Lab stock
pPICZα-*gel*6	pPICZα carrying *gel*6 gene, AOX1 promoter, α‑MF	Recombinant gene expression vector	This study

*E. coli* Top10 was incubated overnight at 37 °C in Luria–Bertani (LB) medium (1 g peptone, 1 g yeast extract, 0.5 g NaCl dissolved in 100 mL deionized water). *E. coli* Top10 Transformer was screened in LB plates containing ampicillin (100 µg/mL).

The yeast transformants (GS115/pPICZα-*gel*6) were screen on YPDZ plates (2 g peptone, 1 g yeast extract, 2 g glucose, and 2 g agar dissolved in 100 mL of deionized water, supplemented with 100 µg/mL Zeocin) (Ma et al. 2022; Xiang et al. 2022).

Yeast was first inoculated on YPD plates (28 °C, 48 h). Subsequently, it was transferred to fermentation medium containing 1% yeast extract, 2% peptone,100 mM potassium phosphate (pH 6.0), 1.34% yeast nitrogen base, 4 × 10^− 5^% biotin, and 1% glycerol (BMGY) or 1% methanol (BMMY) at 28 °C(Ma et al. 2022; Song et al. 2019).

### Design of the human-like recombinant gelatin (hlrGEL6)

When designing hlrGEL6, it is necessary to fully consider its soluble expression in *P. pastoris* and its potential applications in biomedical material scaffolds and cell adhesives. Based on the properties of amino acids, the hydrophilicity of gelatin can be improved by increasing the proportion of polar amino acids in its gelatin amino acid sequence, and the proportion of basic amino acids to provide more positive charges (i.e., higher isoelectric point) to meet its application requirements(Chen et al. 2021; Tian et al. 2005).

The human-like recombinant gelatin (hlrGEL6) is composed of six collagen protein fragments (GEL1) linked together by five GS-linkers, with each GS-linker consisting of 30 amino acids, i.e. 24 glycines and 6 serines (Table 2). The human-like recombinant gelatin (hlrGEL6) is designed to increase its hydrophilicity and isoelectric point, which is determined by the strategy adopted for selecting and designing GEL1. In this case, collagen alpha 1 type III (COL3A1_Human, Uniprot ID: P02461) amino acid sequence (AA: G984-P1028) was chosen as a template to design GEL1. Based on the amino acid composition, proportion and specific sequence analysis of the α1 chain domain, characteristic Gly-X-Y repeat units as the minimum building unit for sequence design. The hydrophobic amino acids (Leu, Pro) in GEL1 were replaced with hydrophilic amino acids (such as Asp, Asn, Glu, and Gln) to enhance the hydrophilicity, while the acidic amino acids (Asp and Glu) were substituted for basic amino acids (Arg and Lys) to increase the isoelectric point of GEL1. After the substitution, there were fewer hydrophobic amino acids (reduced from 11 to 5) and more basic amino acids (increasing from 4 to 7) in comparison with the original sequence. The designed gelatin monomer (GEL1), consisting of 45 amino acids, has a nucleotide length of 135 bp (named gene *gel*1) (Additional file 1).

**Table 2:** List of the gelatin monomer (GEL1) and recombinant gelatin (hlrGEL6)

Name	Amino acid sequence	Sequence size(*N*→C)(AA)
GEL1	GERGDPGSPGNQGQPGNKGSPGPQGPAGQRGNKGERGERGERGAS	45
GS-linker hlGEL6	GGGGSGGGGSGGGGSGGGGSGGGGSGGGGS (GERGDPGSPGNQGQPGNKGSPGPQGPAGQRGNKGERGERGERGAS)6 + (GGGGSGGGGSGGGGSGGGGSGGGGSGGGGS)5 + HHHHHH	30 426

*Notes* hlGEL6: GEL1 + GS-linker + GEL1 + GS-linker + GEL1 + GS-linker + GEL1 + GS-linker + GEL1 + GS-linker + GEL1  + 6xHisTag

The signal peptide (consisting of 81 amino acids) was added at the N-terminus and a His-tag sequence (6×His) at the C-terminus of GEL6 to achieve extracellular secretion and detection of a human-like recombinant gelatin (hlrGEL6). After completing its secretion function, the signal peptide was cleaved by the *KEX*2 enzyme and no longer existed within hlrGEL6. Therefore, hlrGEL6 consists of a total of 426 amino acids with the theoretical molecular weight and the theoretical isoelectric point values of 36.49 kDa and 10.64 respectively. The DNA sequence corresponding to hlrGEL6 (named *gel*6) with a length of 1278 bp was designed in compliance with the codon preference of *P. pastoris*. The amino acid sequence and its corresponding nucleotide sequence have been submitted to the GenBank database with the accession number OR365074.1 obtained.

To meet the requirements for subsequent construction of recombinant vectors, the 5’ end of *gel*6 should be introduced *Xho*I restriction endonuclease recognition site (ctcgag) and *KEX*2 enzyme cleavage site sequence (aaaaga), while the 3’ end should be added with *NotI* restriction endonuclease recognition site (gcggccgc) (Song et al. 2019). Subsequently, the designed nucleotide sequence was transferred to Universal Biotech Co., Ltd. for gene synthesis, which (*gel*6) was successfully synthesized and cloned into the pUC57 vector, and is stored in *E. coli* TOP10 (TOP10/pUC57-*gel*6) (Table 1, Additional file 1).

### Construction of the expressing strain

**Fig. 1:**
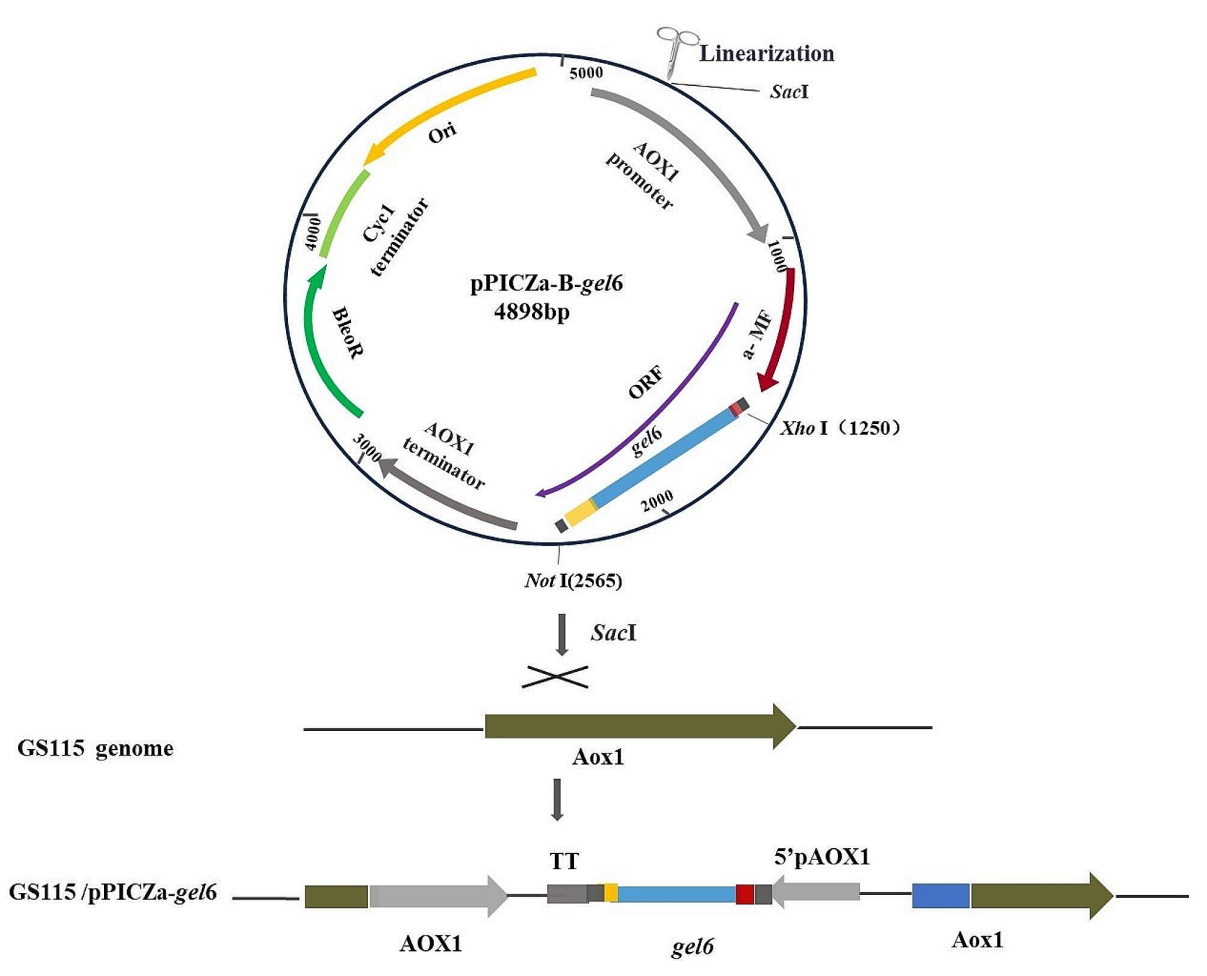
A Sketch map of construction of recombinant *P. pastoris* GS115 with hlrGEL6 gene(*gel*6). The circle graph: Schematic map of the recombinant expression vector pPICZα-*gel*6. The *gel*6 gene was cloned into pPICZa-B using *Xho*I and *Not*I sites as described in Materials and methods

The construction principle of the hlrGEL6 expression strain is illustrated in Fig. 1 and the strain was constructed based on the references (Ma et al. 2022; Xingyin et al. 2023). The target gene (*gel*6) was amplified with *gel*6-F/*gel*6-R primers under the catalysis of DNA polymerase with pUC57-*gel*6 as a template (Additional file 2: Fig. S1, Table S1). After digestion of the target gene (*gel*6) and vector (pPICZa-B) with *Xho*I and NotI enzymes, T4 DNA ligase was used to connect the resultant fragments to generate recombinants, which (pPICZα-*gel*6) were transformed into *E. coli* Top10 cells and sequenced to ensure no mutations occurred in the nucleotide sequence. The correctly ordered transformants were named Top10/pPICZα-*gel*6.

The pPICZα-*gel*6 was extracted from the Top10/pPICZα-*gel*6 and linearized through *Sac* I. Approximately 3 000 ng of linearized plasmid was added into GS115 competent cells and transferred to an electroporation cuvette, in which it was shocked at 2 KV for 5.6 ms. Immediately after the electroporation, 1 mL of pre-cooled 1 mol/L D-sorbitol was added for recovery in a shaker at 28 ℃ for a duration of 2 h. The recovered cells were then spread evenly on YPDZ plates (Zeocin:200 µg/mL) and, after mono-clone growth, 20 clones were randomly selected for PCR amplification to determine whether the transformation was successful. Ten clones (GS115/pPICZα-*gel*6) identified as positive were then selected and inoculated into culture dishes containing 2 mL of BMGY medium to be cultured at 28 ℃ until the *OD*_600_ reached 5 ~ 6, after which BMMY medium containing 1% methanol was used for induction. The suspended solution was then cultured at 28 ℃ for 48 h to allow for the collection of the supernatant for detection of recombinant protein expression. SDS-PAGE and Western blot techniques were employed to detect the secretion of proteins, with a commercial antibody (anti-His-Tag-HRP) being used for Western blot analysis. The single clone with the highest expression level was selected for cultivation and protein production in shake flasks (Song et al. 2019).

### Expression and purification process of hlrGEL6

The GS115/pPICZα-*gel*6 (4# clone) with the highest expression level was inoculated into 5mL of YPD liquid medium and cultured overnight in a shake flask. Subsequently, the culture was transferred to 50 mL of BMGY (pH 6.0) and incubated at 28℃ and 200 rpm for 24 h to measure *OD*_600_. Once the *OD*_600_ reached 6, the strains were collected by centrifugation (3 000 g, 5 min), suspended in 200 mL of BMMY (methanol 1%, pH 6.0) for induction expression (initial *OD*_600_ = 1.0 ~ 1.5), which lasted for a duration of 72 h at 28 ℃ with the addition of methanol (V/V) once every 24 h.

The pH of the fermentation supernatant was adjusted to 7.4 followed by centrifugation at 12 000 g for 30 min to remove yeast precipitation and obtain the supernatant. After filtration through a 0.45 μm filter membrane, it was injected into a nickel column in an AKTA protein purification system and purified in accordance with the reference (Song et al. 2019). The steps were performed as follows: equilibration (50 mmol/ L PBS, 0.3 mol/L NaCl, pH 7.4) → sample loading → equilibration(50 mmol/L PBS, 0.3 mol/L NaCl, pH 7.4) → washing (50 mmol/L PBS, 0.3 mol/L NaCl, 20 mmol/L imidazole, pH 7.4) → elution (20 mmol/L PBS, 0.3 mol/L NaCl, 300 mmol/L imidazole, pH7.4), and the eluate was collected. The eluate was dialyzed with PBS (pH 7.4) to remove imidazole, and the purified protein solution (12 mL) was obtained. Non-reducing SDS-PAGE electrophoresis analysis was used to analyze the purified samples with a gel concentration of 12%, and Western Blotting technology was used to identify target proteins using His-Tag-HRP antibody. SDS-PAGE and Western blot analysis were performed on both the induced culture supernatants and the intracellular products to determine whether the target protein was secreted extracellularly. Protein concentrations were measured with a Bradford Protein Assay Kit (TaKaRa) with bovine serum protein (Thermo) for the standard protein(Nouroozi 2015) .

### Amino acid analysis of hlrGEL6

The aqueous solution of pure hlrGEL6 was neutralized with NaOH following the hydrolysis with 6 mol/L HCl at 120 ℃ for 22 h. Subsequently, the samples were derivatized with phenyl isothiocyanate (PITC) to obtain free amino acids, and their contents were analyzed with high performance liquid chromatography (HPLC) (LC-20AD, Shimadzu, Japan). The resultant raw data were automatically integrated with Labsolution software through the external standard method, and the molar percentage of amino acid composition in the sample was calculated based on the corresponding peak area (Betnér 1986).

#### Molecular weight determination of hlrGEL6

The hlrGEL6 aqueous solution (1 µL) was mixed with a matrix solution (0.6 µL) containing saturated α-cyano-4-hydroxycarboxylic acid, 50% acetonitrile, 0.1% trifluoroacetic acid, and 10% acetone. The mixture was then spotted onto a steel MALDI plate and allowed to dry, after which the relative molecular weights were determined with matrix-assisted laser desorption ionization time-of-flight mass spectrometry (ABSciex 5800 MALDI-TOF/TOF, Bruker Daltonics, America). The linear method in the positive ion mode was selected for analysis, and a laser source with a wavelength of 335 nm was used to calibrate the sample against a test standard to obtain accurate molecular weight measurements within the scanning range of 1000 ~ 80,000 Da. Finally, flexAnalysis software was used to analyze and process the raw data and spectra generated by MALDI-TOF/TOF(Hansen and Lee 2018; Honeker et al. 2022).

#### Self-crosslinking of hlrGEL6

The purified recombinant collagen was concentrated to not less than 1.5 mg/mL through ultrafiltration (3500 rpm, 30 min), and then adjusted to pH 9.5 with a 0.01-fold volume of 5.0 mol/L NaOH solution. Subsequently, 200 µL of the aforementioned solution was transferred into a 2.0 mL Eppendorf tube and left undisturbed at room temperature for not less than 30 min to observe hydrogel formation. The same method was used in the controlled experiment with commercially available collagen (by Shanghai Chemical Reagent Company).

### Analysis of hlrGEL6 hydrogel structure

The internal pore distribution and characteristics of the samples were determined with a scanning electron microscopy (SEM) (TESCAN MIRA LMS, Czech Republic). To preserve the intact 3D porous structure of the gel, the hydrogel samples, after being freeze-dried in liquid nitrogen, were meticulously sectioned with a cold knife into thin slices, which were placed on copper substrates and observed under a scanning electron microscope after being coated with gold (Novikov 2023; Venkateswarlu et al. 2020). The control experiment was conducted using the same method.

### 3D cell culture of hlrGEL6 hydrogel

First, the hlrGEL6 gelatin solution was prepared by the aforementioned experimental method, transferred into a 96-well plate with 150 µL per well, and left undisturbed at room temperature until the hlrGEL6 hydrogel formed. Subsequently HepG2 cells was digested with a 0.25% trypsin-EDTA solution and suspended in DMEM medium containing 10% FBS to obtain a cell suspension before 150 µL of HepG2 cell suspension was seeded onto each hydrogel microsphere to ensure the cell density of 5000 per well (Egger et al. 2024). Finally, the 3D-cells (named hlrGEL6-HepG2 group) were incubated for 48 h with the culture medium changed every day. Two control groups were also set up: one without the use of gelatin (named HepG2 group) and the other with the use of commercially available gelatin as the cell culture scaffold (named gelatin-HepG2). The cell seeding amount and culture conditions of the control groups are identical to those of the experimental group.

After 48 h of incubation, the Calcein-AM/PI dual staining kit was used to assess cell viability and proliferation. The specific experiment procedures were conducted in conformity with the instructions of the Calcein-AM/PI dual staining kit. Firstly, the hydrogel-cell complex were gently washed with a PBS buffer solution to remove any excess culture medium. Then, the hydrogel-cell complex were stained with Calcein-AM and PI until they were fully colored, and subsequently placed in PBS to eliminate any remaining Calcein-AM/PI residue. After dyeing, a Leica DMi8 confocal laser scanning microscope (CLSM) was used to observe the fuorescent HepG2 cells. For the control group (the Gelatin-HepG2), cell fluorescence signals were observed with CLSM (LSM 980, Zeiss).

## Results and discussion

### Recombinant vectors construction and transformation

**Fig. 2:**
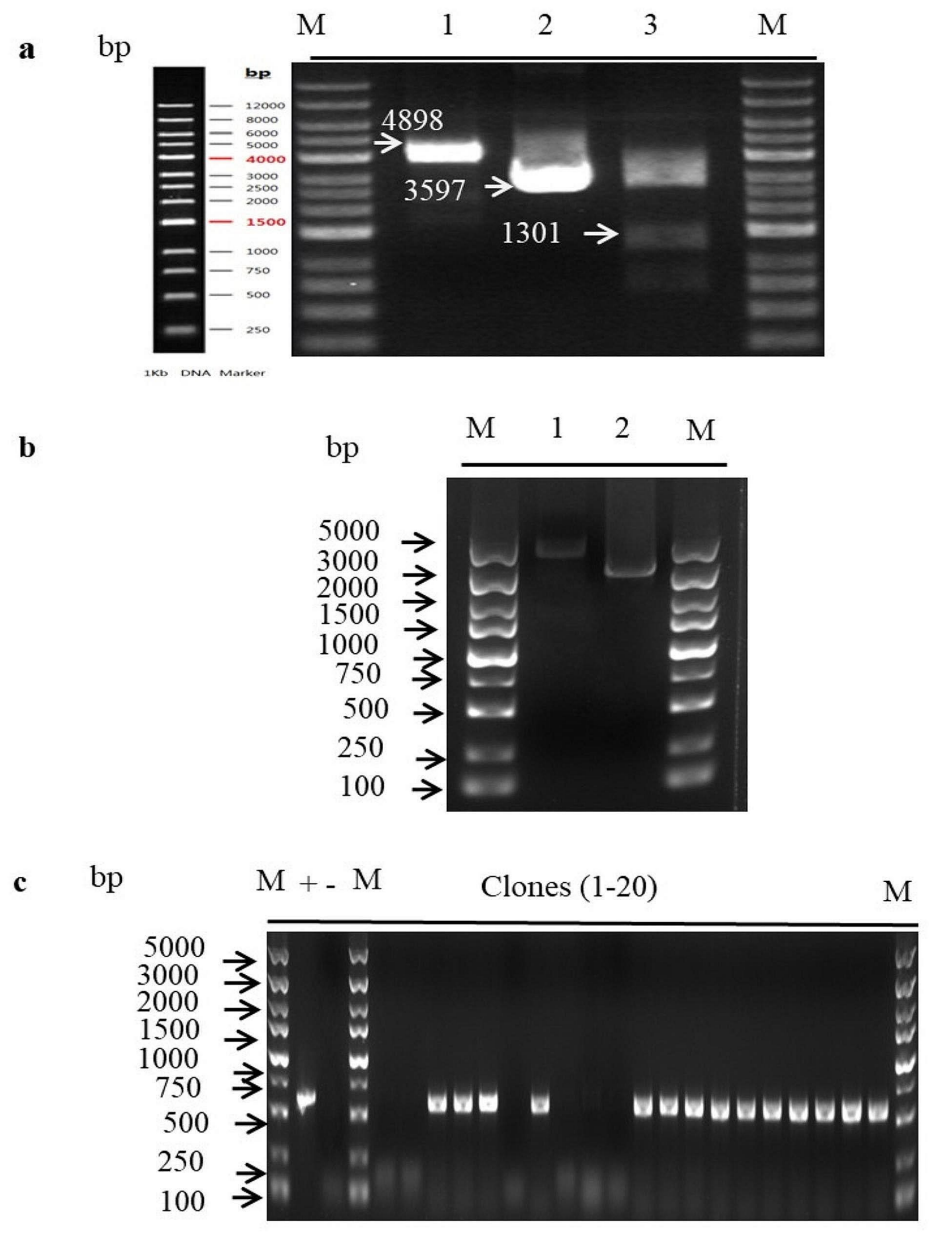
Construction and transformation of recombinant vectors. (**a**) Identification of pPICZα-*gel*6 recombinant vector by double digestion, M: DNA marker, 1: Recombinant, 2: empty vector. 3. Double enzyme digestion vector. **(b)** Linearization of pPICZα-*gel*6, M: DNA marker, 1: before linearization, 2: after linearization. (**c)** Electrophoretic detection of PCR products of transforming strain, M: DNA marker, 1–20: PCR identification of different clones, - : negative control (empty vector), +: positive control (recombinant vector)

The expression vector pPICza-B-*gel*6 was extracted for double digestion with *Xho* I and *Not* I to yield two DNA bands that matched the expected sizes (1301 bp and 3597 bp), confirming the correct construction of the expression vector containing the *gel*6 gene (Fig. 2a). Afterwards, this properly constructed vector was linearized and introduced into GS115 for transformation. The results of pPICZα-*gel*6 linearization are shown in Fig. 2b, demonstrating successful linearization. Subsequently, the linearized vector was electro-transformed into GS115 and plated on YPDZ medium supplemented with 200 µg/mL Zeocin, followed by incubation at 28 ℃ for 2 ~ 3 days. After monoclonal growth, a total of 20 clones were randomly selected for PCR amplification. It was confirmed through agarose gel electrophoresis analysis that the presence of an amplified fragment complied with the theoretically predicted size of 540 bp (Fig. 2c).

**Fig. 3:**
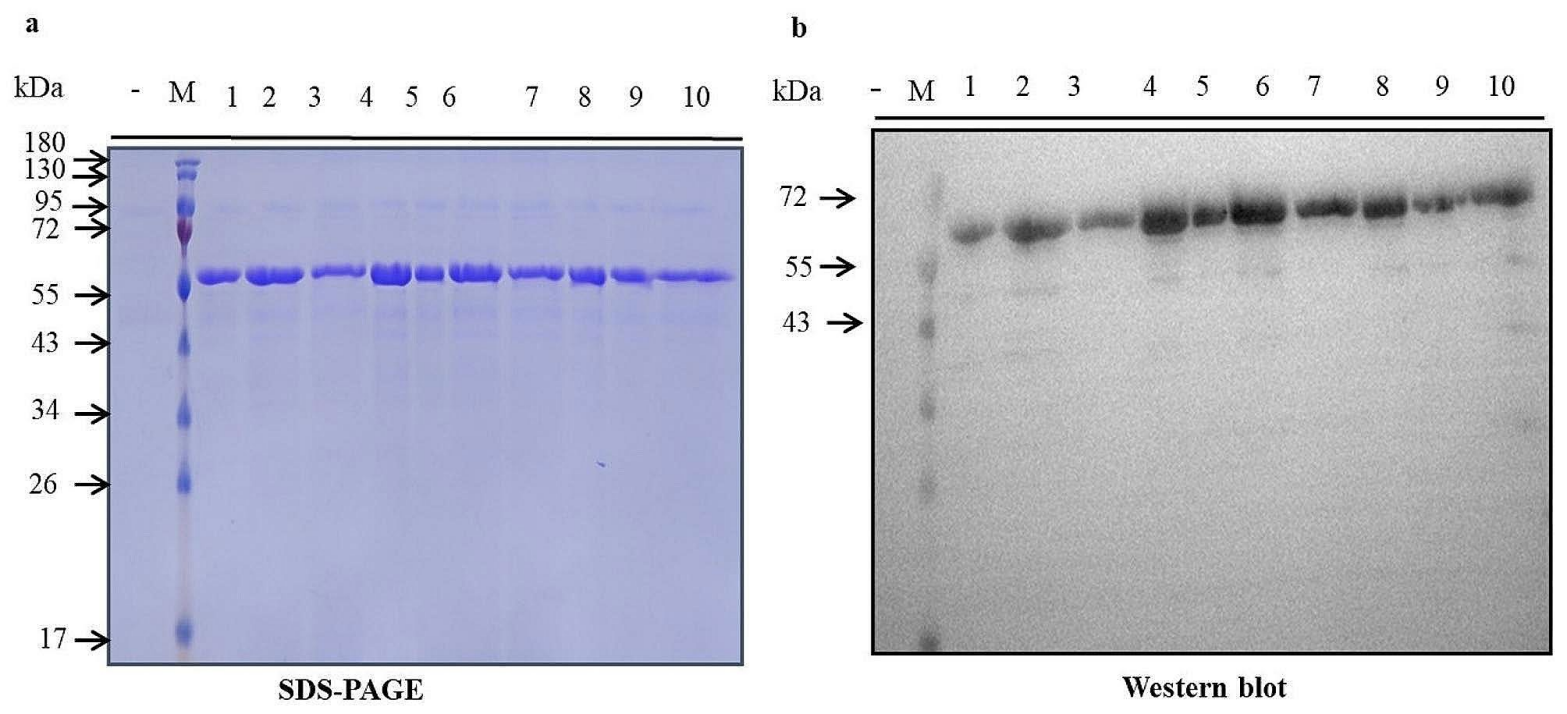
SDS–PAGE and Western blot analysis of hlrGEL6 prepared by small shake tubes (**a**) SDS-PAGE was used to detect the culture supernatant. (**b**) Western blot detection of the culture supernatant(Anti-His-HRP). M: Protein marker, 1–10: positive colonies, -:Negative control (non-reductive)

Ten single colonies were randomly chosen from these identified clones and inoculated into a culture medium (2 mL) for induction and cultivation for 48 h, after which protein secretion and expression were assessed through SDS-PAGE and Western blot techniques. According to the SDS-PAGE electrophoresis results shown in Fig. 3, a total of ten clones successfully expressed the target protein, but their expression levels varied. The variation in protein expression levels is attributed to random integration of the recombinant vector into the AOX1 region of GS115 genome after linearization, resulting in different gene dosages for each transformant. Our previous studies have confirmed a positive correlation between increased gene copy number and enhanced production of the target protein. Among them, clone (4#) exhibited higher expression levels and was subsequently utilized for shaking flask fermentation (Fig. 3).

### Expression and purification of hlrGEL6

**Fig. 4:**
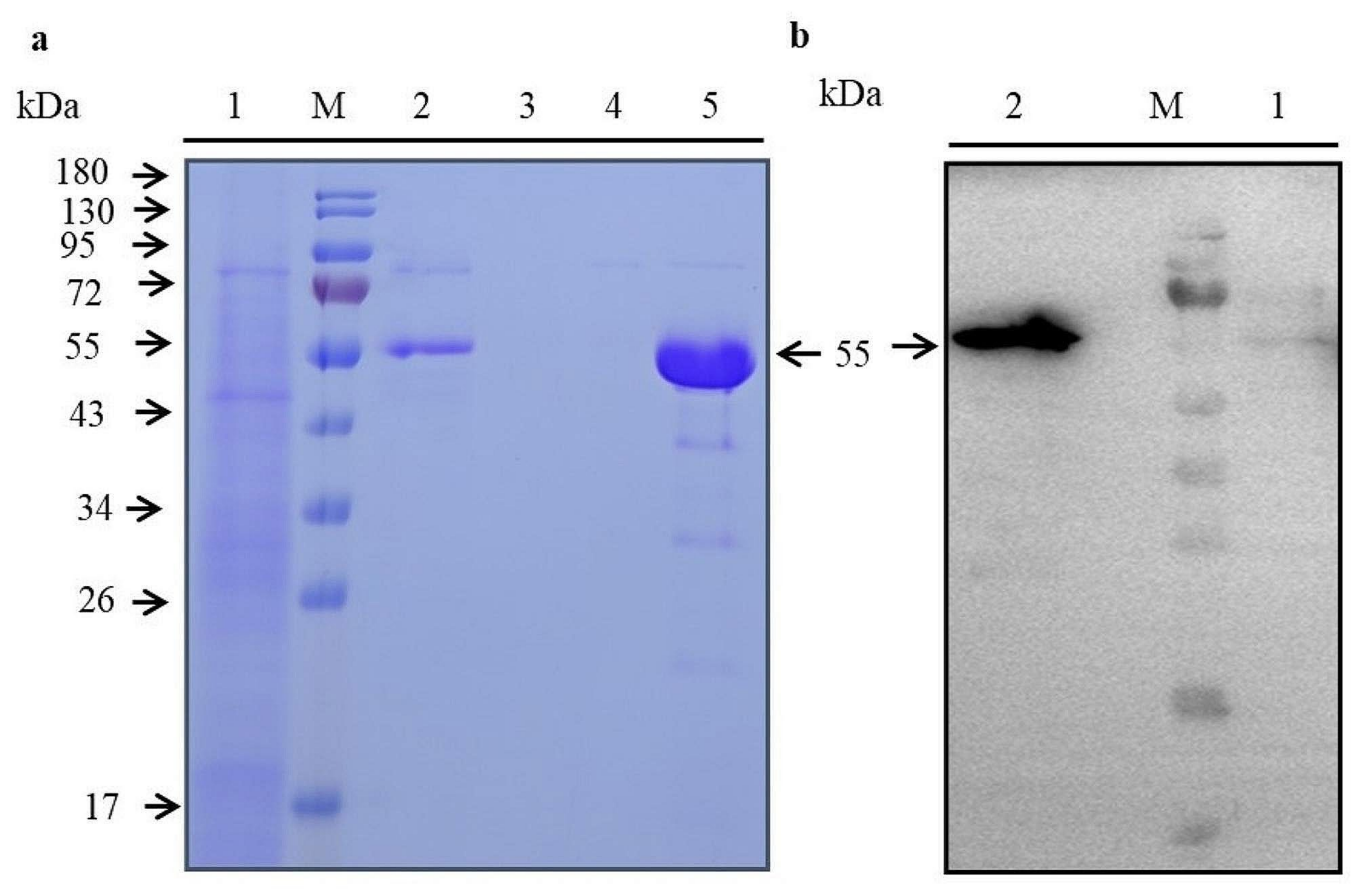
Analysis of the purification results of hlrGEL6 prepared by shake flask fermentation (**a**) The purified protein was detected by SDS-PAGE. (**b**) The protein secretion was detected by Western blot. M: protein marker, (1) Intracellular sample after induction, (2) Fermentation supernatant sample, (3) effluent sample, (4) cleaning fluid sample, (5) elution sample

The clone with the highest expression level was selected for shake flask cultivation (cultivation system of 200 mL), and fermentation was terminated after 72 h of induction. The fermented product was purified through affinity chromatography, resulting in 12 mL high-purity hlrGEL6, of which hlrGEL6 purification and protein secretion were assessed with SDS-PAGE and Western blot respectively (Fig. 4). According to the results of SDS-PAGE, only one protein band with a molecular weight of 55 kDa was observed in the elution sample, indicating high protein purity (Fig. 4a). Additionally, based on the Western blot analysis of both the cultured supernatant and induced intracellular products, it was discovered that most of the target proteins were secreted into the fermentation supernatant with a small fraction remaining within the cells (Fig. 4b), while the purified protein concentration was determined 0.95 mg/mL as through BCA assay. These findings indicated that almost all of the designed hlrGEL6 in this study were successfully secreted into the extracellular space through *P. Pastoris*. Furthermore, it is worth noting that 11.4 mg of purified protein (hlrGEL6) was obtained in a culture volume of 200 mL, which is equivalent to a fermentation yield of 0.057 mg/mL. Although the recombinant strain (4#) currently exhibits a relatively high expression level, there is still potential for further enhancement compared to the yields reported in previous literature(Wang et al. 2014; Xiang et al. 2022). It is planned to modify and optimize the existing strain by means of antibiotic high-pressure screening, promoter engineering, fermentation conditions optimization, etc. for the purpose of obtaining an engineered strain of higher expression levels.

### Amino acid analysis of hlrGEL6

The analysis of amino acid composition can be utilized to comprehend the chemical structural characteristics of polypeptide proteins and provide scientific insights into protein structure and function. The results revealed that the observed number and molar percentage of amino acids in hlrGEL6 are within theoretical expectations as shown in Table 3 (Additional file 3: Fig. S2). Due to limitations in acid hydrolysis techniques and the resolution capabilities of automated amino acid analyzers, only nine major amino acids were presented in the table (Table 3). However, it is sufficient to demonstrate that the detected values of recombinant gelatin match their theoretical values. Compared to other types of proteins, both human type III α-collagen chains and recombinant gelatin have relatively high levels of glycine and proline content, which is a distinct feature that distinguishes them from other proteins.

**Table 3:** Amino acid analysis of hlrGEL6

Amino acid	AA number of theories	Measured sample			
		AA mole	AA number	Error (%)	Mole (%)
Asp	24	921.92 ^a^	23.64 ^a^	-1.49^a^	5.21 ^a^
Glu	48	1800.43 ^b^	46.17^b^	-3.81^b^	10.17^b^
Ser	48	1769.81	45.39	-5.44	10.00
Gly	210	9147.96	234.61	11.72	51.67
His	6	269.15	6.90	15.04	1.52
Arg	30	1234.96	31.67	5.57	6.98
Thr	0	-	-	-	-
Ala	12	506.95	13.00	8.34	2.86
Pro	36	1538.87	39.47	9.63	8.69
Tyr	0	-	-	-	-
Val	0	-	-	-	-
Met	0	-	-	-	-
Cys	0	-	-	-	-
Ile	0	-	-	-	-
Leu	0	-	-	-	-
Phe	0	-	-	-	-
Trp	0	-	-	-	-
Lys	12	513.99	13.18	9.85	2.90
Total	426	100	426^c^		

-: Not detected

^a, b^ Asn and Gln will generate Asp and Glu, respectively, after acid hydrolysis treatment, so the values in the table are Asn + Asp and Gln + Glu

cThe detection value is calculated based on the theoretical number and molecular weight of amino acid residues in reconstituted collagen

### Molecular weight determination of rhGEL6

Molecular mass is a crucial characteristic parameter of protein samples and is essential for the identification and subsequent research of new proteins. In this study, the theoretical molecular weight of hlrGEL6 was determined to be 36.49 kDa; however, an apparent molecular weight ranged from 43 to 55 kDa for the target protein as shown in SDS-PAGE results (Figs. 3 and 4). To investigate the reason for the discrepancy between the apparent molecular weight and theoretical molecular weight of hlrGEL6, matrix-assisted laser desorption ionization time-of-flight mass spectrometry (MALDI-TOF-MS) was utilized to analyze purified samples in order to determine their precise molecular weights. As a novel soft ionization technique developed in recent years, MALDI-TOF-MS introduces matrix molecules to prevent the fragmentation of non-volatile and thermally unstable biomolecules during the ionization process, hence enjoying significant advantages in determining the molecular weight of biomolecules and synthetic polymers.

**Fig. 5:**
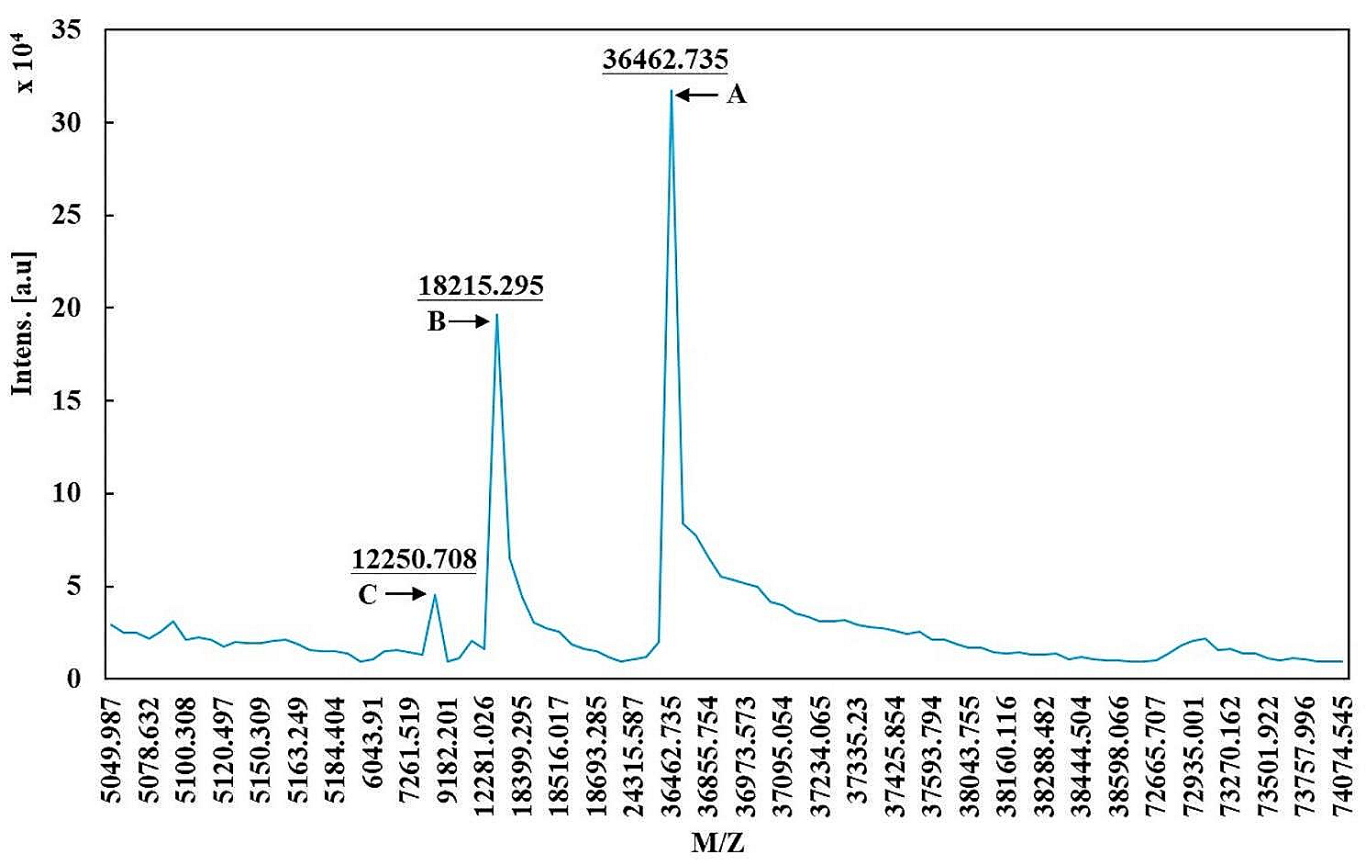
Molecular weight of hlrGEL6

As shown in Fig. 5, the experimental results indicated the presence of a distinct protein peak (indicated by arrow A) with a molecular weight of 36462.735 Da, presumably a recombinant gelatin monomer carrying one ion charge, in addition to two other peaks observed (indicated by arrow B and C), which could be ion fragment peaks. It was revealved through MALDI-TOF-MS analysis that the accurate molecular weight (36.46 kDa) of hlrGEL6 is identical to its theoretical molecular weight (36.49 kDa).

The hlrGEL6 designed theoretically consists of 426 amino acids, with an apparent molecular weight of 43 ~ 55 kDa. However, the actual precise molecular weight (36.46 kDa) is slightly larger than the theoretical one. The significant difference between the apparent and actual molecular weights of hlrGEL6 can be attributed to the presence of Gly-X-Y composition in this gelatin. Based on previous observations from recombinant protein expression studies(Butkowski et al. 1982), we have found that this difference is also common in other target proteins where the apparent molecular weight is usually larger than the theoretical value. This phenomenon can be reasonably explained by gelatin containing a large number of hydrophilic residues, leading to reduced affinity for SDS binding and decreased negative charge on targeted proteins. Therefore, they migrate slower and have shorter migration distances in SDS-PAGE. To verify whether the band truly represents the target protein, Western blotting analysis was conducted, in which no expression signal was observed in the control group according to the results presented in Fig. 3a, but a distinct 55 kDa band was identified in the experimental group, providing evidence for its correlation with our expected target protein.

### Gelation of hlrGEL6

By adjusting the pH of the hlrGEL6 solution, gelation can be achieved. Gelation generally refers to the gradual thickening and eventual loss of fluidity of certain solutions during cooling. The molecules or particles dispersed in the solution gradually aggregate to form a three-dimensional network structure, thereby generating hydrogel (Sun et al. 2024). After being left undisturbed for over 30 min, a hydrogel formed at the bottom of the Eppendorf tube containing 200 µL of hlrGEL6 solution (Additional file 4: Fig.S3a). In comparison to commercially available gelatin, the hlrGEL6 hydrogel has a lower minimum gelation concentration of 1.5 mg/ml, whereas the minimum concentration for commercially available gelatin is 2.0 mg/ml (equivalent to 0.2%) (Additional file 4: Fig.S3b).

### SEM analysis of hlrGEL6

**Fig. 6:**
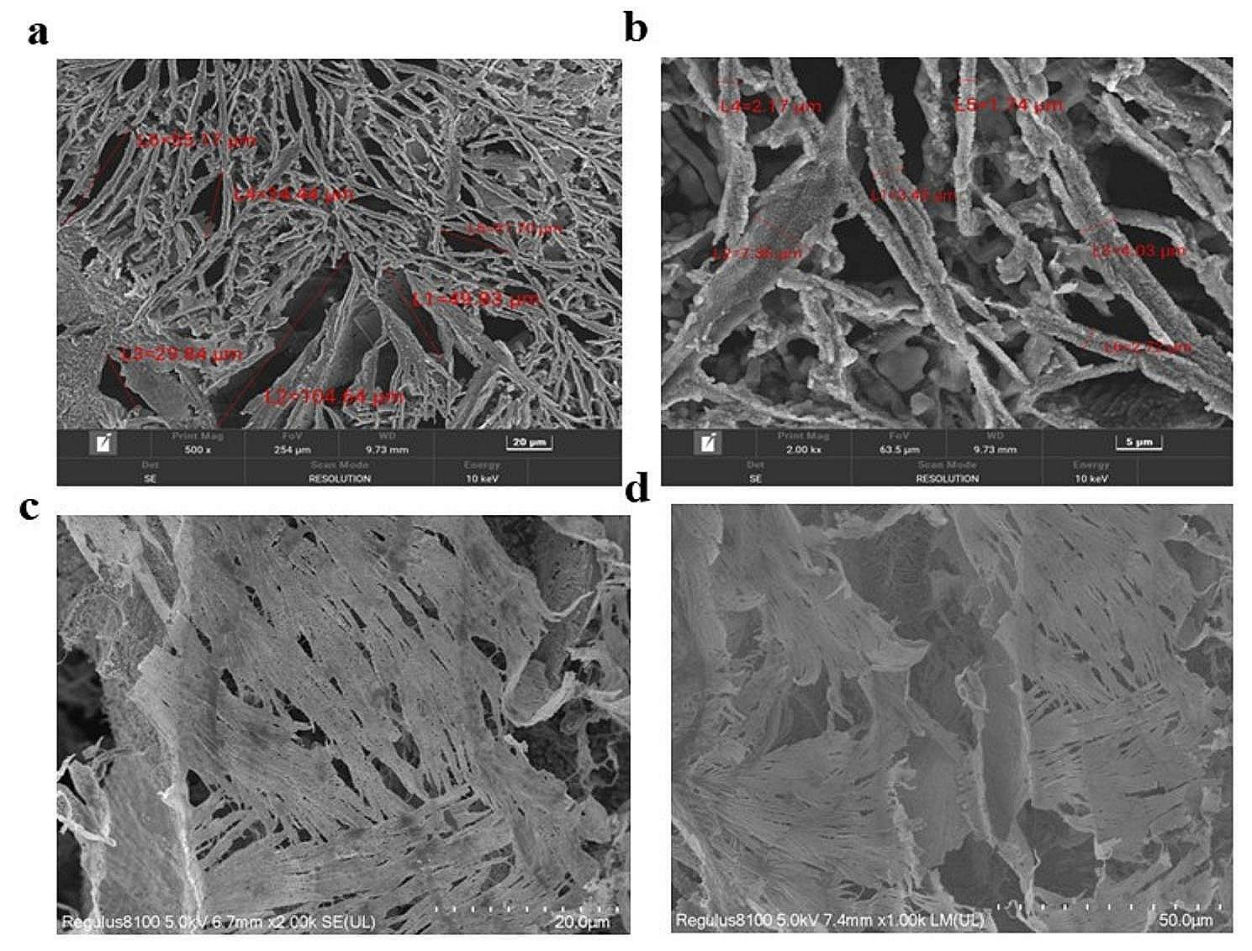
SEM analysis of hlrGEL6 **(a)** The porous structure of hlrGEL6. **(b)** Diameter of the hlrGEL6. **(c)** The porous structure of commercially available gelatin. (d) The diameter of commercially available gelatin

The Fig. 6 shows two scanning electron microscope (SEM) spectra with magnifications of 500 or 2000 times, demonstrating the clearly visible porous structure in hlrGEL6 hydrogel, with the main pore size distribution ranging from 30 ~ 70 μm and a maximum pore size of up to 100 μm. Additionally, the average diameter of the hydrogel is approximately 2 ~ 3 μm (Fig. 6a, b). In comparison to commercially available gelatin, the hlrGEL6 hydrogel exhibits a more uniform pore distribution and larger fiber diameter (Fig. 6c, d). In general, hydrogels exhibit a sponge-like structure, indicating their potential as biomedical materials.

### The 3D cell culture of the hlrGEL6 hydrogel

After 48 h of cultivation, the Calcein-AM/PI dual staining kit instructions were followed to perform dual staining to observe the morphology of HepG2 cells. The laser scanning confocal microscope (LSCM) was utilized with an emission wavelength of 490 nm and excitation wavelengths of either 515–617 nm.

**Fig. 7:**
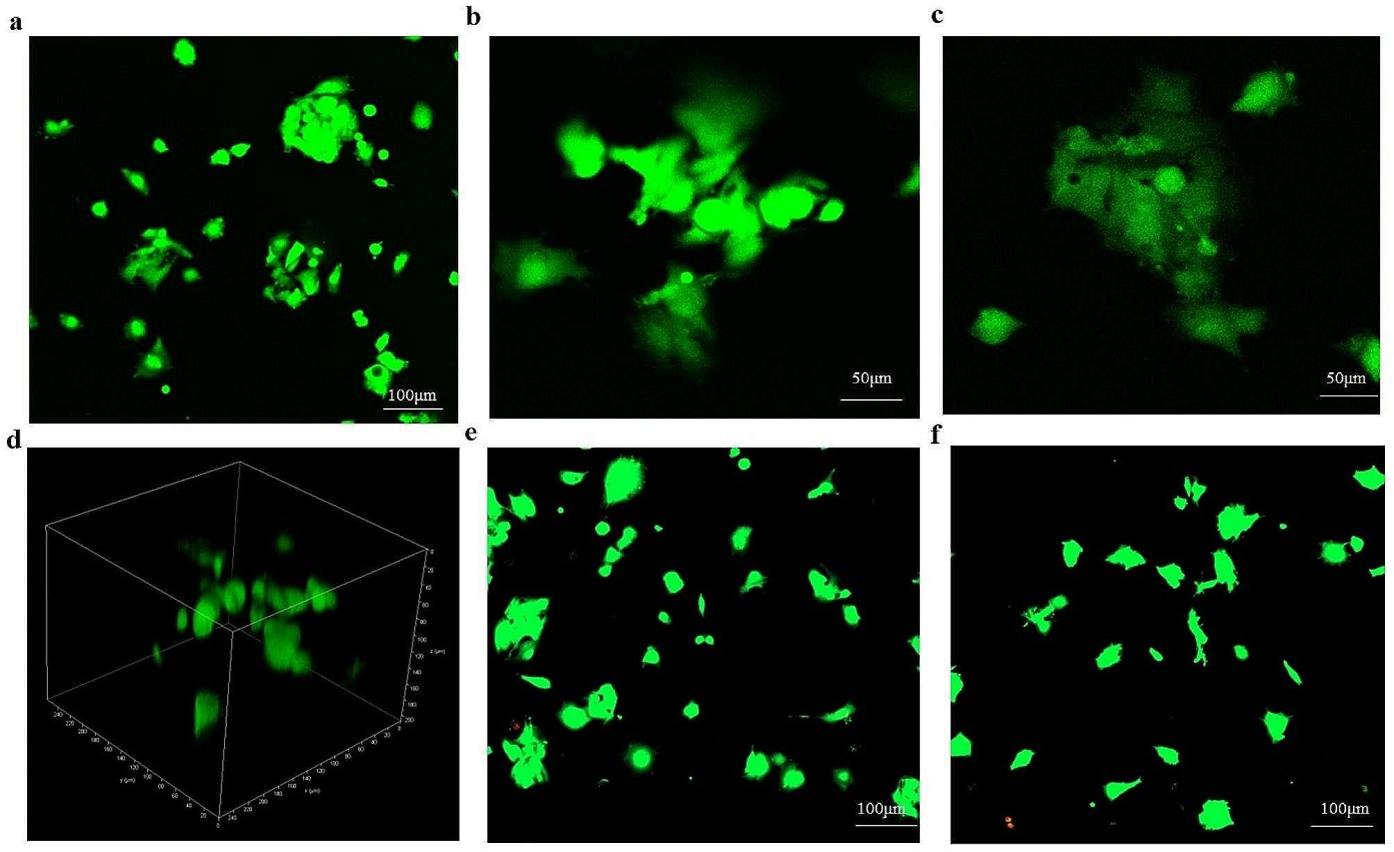
Three-dimensional cell culture of hlrGEL6 hydrogel (**a-d**) hlrGEL6-HepG2 group. (**a**) The cells cultured in hlrGEL6 hydrogel appear rounded and form clusters. (**b-c**) The are one of the cell aggregates that are formed in hlrGEL6 hydrogel culture. (**d**) This is a screenshot of video images displayed HepG2 cells cultured in hlrGEL6. (**e**) gelatin-HepG2 group. (**f**) HepG2 group

The fluorescence staining results of Calcein-AM/PI showed that the cells in the hlrGEL6-HepG2 group mainly appeared round and formed aggregates. All observed cells exhibited green fluorescence signal, without any red fluorescence signal (Fig. 7a-d). In comparison, cells in the gelatin-HepG2 group appeared round or spindle-shaped, with some cells able to aggregate (Fig. 7e). The HepG2 group demonstrated the characteristic spindle shape of two-dimensional culture (Fig. 7f). These experimental results indicate that using recombinant gelatin as a three-dimensional culture scaffold can significantly improve cell survival rate compared to the gelatin-HepG2 group.

The solubilities of collagen α1 chain III, the hexamer of the unmodified monomer, and hlrGEL6 were predicted with an online software for protein solubility prediction (https://www.novopro.cn/tools/prot-sol.html). As shown in theoretical calculations, their solubilities were 0.530, 0.452, and 0.496 respectively (Additional file 5). It was demonstrated through the prediction results that the water solubility of hlrGEL6 is similar to that of natural gelatin and slightly higher than that of the hexamer of the unmodified monomer. It is widely accepted that, in case of the protein solubility higher than 0.45, dissolution seems to be easier compared with the average value in Niwa et al.‘s dataset from 2009 (Niwa et al. 2009).

Additionally, it possesses more positive charge characteristics for the proximity of its theoretical isoelectric point (pI) to that of poly-lysine used for adhesive coating agents, hence promoting cell adhesion during cultivation and improving cultured cell survival rate. Furthermore, hlrGEL6 also exhibits self-crosslinking ability which enables it to form a unique porous network structure in three-dimensional hydrogels. These features indicate its potential as a biomedical material and lay a foundation for its application in tissue engineering.

## Conclusions

The *P. pastoris* expression system successfully enabled soluble secretion expression of hlrGEL6, which contains multiple repeated peptide monomers (GEL1). At the shake flask fermentation level, the expression level reached 0.057 g/L. Compared with the reported expression level of the same strain in recent literature (0.032 g/L), the original strain showed a higher expression level as it was possible to obtain up to 11.4 mg of high purity protein in a 200 mL culture system(Xiang et al. 2022). The pH adjustment of the hlrGEL6 solution enabled it to spontaneously form a uniform porous structure. When hlrGEL6 hydrogel was used as a scaffold for HepG2 cell culture, obvious characteristics of 3D cell culture were demonstrated with better cell viability, highlighting the great potential as a biomedical scaffold material. No industrial-level high-efficient expression, however, has been achieved with the recombinant expression strains (GS115/pPICZα-*gel*6,4#) currently constructed. The follow-up research plans to improve this strain by antibiotic high-pressure screening, promoter engineering, fermentation condition optimization, etc. in order to obtain an engineered strain suitable for mass production and increase hlrGEL6 production to be developed into biomedical materials.

### Electronic supplementary material

Below is the link to the electronic supplementary material.

Additional file 1: The expression cassette sequence of hlrGEL6 and its gene. sequence

Additional file 2:Table S1, Fig. S1: Amplification of target gene (*gel*6)

Additional file 3: Additional file3: Fig. S2 Liquid chromatography of hlrGEL6

Additional file 4: Additional file 4: Gelation of hlrGEL6

Additional file 5: Additional file 5: Protein sequences of solubility prediction

Additional file 6

## Data Availability

All data generated or analyzed during this study are included in this published article.

## Abbreviations

hlrGEL6: Type III human‑like gelatin
MALDI‑TOF‑MS: Matrix‑assisted laser desorption ionization time‑of‑flight mass spectrometry
SEM: Scanning electron microscopy
3D: Three-dimensional cell culture
LSCM: Laser scanning confocal microscopy
