# Supplementary material for: Expression, characterization, and application of human-like recombinant gelatin
Source: Bioresour Bioprocess. 2024 Jul 17;11(1):69. doi: 10.1186/s40643-024-00785-1 (PMC11252100; doi:10.1186/s40643-024-00785-1)
Supplement: Supplementary file 1 — Additional file 1: The expression cassette sequence of hlrGEL6 and its gene. sequence [file 40643_2024_785_MOESM1_ESM.docx]

**Additional file1**

**The expression cassette sequence of hlrGEL6 and its gene sequence (*gel*6)**

**1. The expression cassette sequence of hlrGEL6 （N → C）(426AA)**

MRFPSIFTAVLFAASSALAAPVNTTTEDETAQIPAEAVIGYSDLEGDFDVAVLPFSNSTNNGLLFINTTIASIAAKEEGVSLEKRGERGDPGSPGNQGQPGNKGSPGPQGPAGQRGNKGERGERGERGASGGGGSGGGGSGGGGSGGGGSGGGGSGGGGSGERGDPGSPGNQGQPGNKGSPGPQGPAGQRGNKGERGERGERGASGGGGSGGGGSGGGGSGGGGSGGGGSGGGGSGERGDPGSPGNQGQPGNKGSPGPQGPAGQRGNKGERGERGERGASGGGGSGGGGSGGGGSGGGGSGGGGSGGGGSGERGDPGSPGNQGQPGNKGSPGPQGPAGQRGNKGERGERGERGASGGGGSGGGGSGGGGSGGGGSGGGGSGGGGSGERGDPGSPGNQGQPGNKGSPGPQGPAGQRGNKGERGERGERGASGGGGSGGGGSGGGGSGGGGSGGGGSGGGGSGERGDPGSPGNQGQPGNKGSPGPQGPAGQRGNKGERGERGERGASHHHHHH

**Note:**

Fluorescent green indicates signal peptides.

Light blue represents six histidine residues.

The section without a colored background represents the target protein sequence, while the light brown background indicates the GS-linker.

**2. The optimized gene sequence of f hlrGEL6 （5^′^→3^′^）**

**(Total length 1301bp, *gel*6 1260bp）**

ctcgagaaaagaGGTGAAAGAGGTGATCCAGGTTCTCCAGGTAATCAAGGTCAACCTGGTAACAAGGGTTCTCCTGGTCCACAAGGTCCAGCTGGTCAAAGGGGTAACAAAGGTGAACGTGGTGAGAGGGGTGAGCGTGGTGCTTCTGGTGGTGGTGGATCTGGTGGCGGAGGTAGCGGAGGTGGTGGTAGTGGTGGCGGTGGTTCAGGCGGTGGTGGCAGCGGAGGCGGAGGATCAGGTGAAAGGGGAGATCCCGGAAGTCCTGGAAATCAAGGACAGCCAGGCAACAAAGGATCTCCAGGACCTCAAGGACCTGCTGGACAACGTGGAAACAAAGGCGAAAGGGGCGAACGTGGCGAGCGTGGCGCTAGTGGCGGTGGCGGTTCTGGCGGAGGTGGATCCGGCGGAGGCGGTTCAGGTGGTGGCGGCTCCGGTGGTGGTGGCAGTGGCGGTGGTGGCTCTGGTGAGAGAGGTGACCCTGGAAGCCCAGGTAACCAGGGACAGCCCGGAAACAAGGGCAGTCCAGGTCCTCAGGGTCCTGCAGGTCAGAGAGGCAACAAGGGCGAGAGAGGTGAGAGAGGCGAGAGGGGCGCATCAGGTGGCGGCGGTAGTGGTGGTGGCGGAAGCGGAGGCGGTGGTTCCGGTGGCGGCGGATCTGGCGGCGGTGGCTCAGGTGGCGGAGGCTCAGGCGAACGTGGTGATCCTGGATCTCCTGGAAACCAGGGCCAACCAGGTAACAAAGGCTCACCCGGACCACAGGGACCCGCAGGACAAAGGGGCAACAAAGGCGAGCGTGGCGAAAGAGGTGAAAGGGGCGCTAGCGGTGGCGGAGGTTCAGGTGGCGGTGGTAGTGGCGGAGGTGGAAGTGGCGGCGGAGGAAGCGGTGGCGGAGGATCCGGCGGTGGTGGTTCCGGCGAAAGGGGTGATCCTGGTAGCCCCGGAAATCAGGGACAACCTGGCAACAAGGGCTCACCAGGTCCTCAAGGCCCCGCAGGCCAGCGTGGCAACAAAGGTGAGAGAGGTGAAAGGGGCGAACGTGGTGCAAGTGGTGGTGGTGGCTCCGGCGGAGGTGGAAGCGGTGGTGGTGGTTCTGGTGGTGGCGGTTCAGGCGGTGGCGGTTCTGGTGGCGGAGGTTCTGGTGAAAGAGGCGATCCTGGCTCACCTGGCAATCAGGGTCAGCCTGGAAACAAAGGCTCCCCTGGTCCTCAGGGCCCAGCCGGACAACGTGGTAACAAGGGTGAGCGTGGCGAACGTGGTGAGAGAGGCGCTTCTCATCATCACCATCACCATTAAgcggccgc

**Note:**

The bright yellow background indicates the *Xho*Ⅰ restriction enzyme cutting site (ctcgag) at the 5' end of *gel*6 and the *Not*Ⅰ restriction enzyme cutting site (gcggccgc) at its 3' end.

The bright blue background indicates the KEX2 enzyme cleavage site sequence (aaaaga) at the 5' end of *gel*6.

The light blue background (CATCATCACCATCACCAT) represents the nucleotide sequence containing six histidine residues.

Red background indicates the termination codon (TAA).

The parts without a colored background represent the target gene sequence (*gel*6).
