# Supplementary figures and images for: Expression, characterization, and application of human-like recombinant gelatin
Source: Bioresour Bioprocess. 2024 Jul 17;11(1):69. doi: 10.1186/s40643-024-00785-1 (PMC11252100; doi:10.1186/s40643-024-00785-1)

**Additional file3** **Liquid chromatography of hlrGEL6**


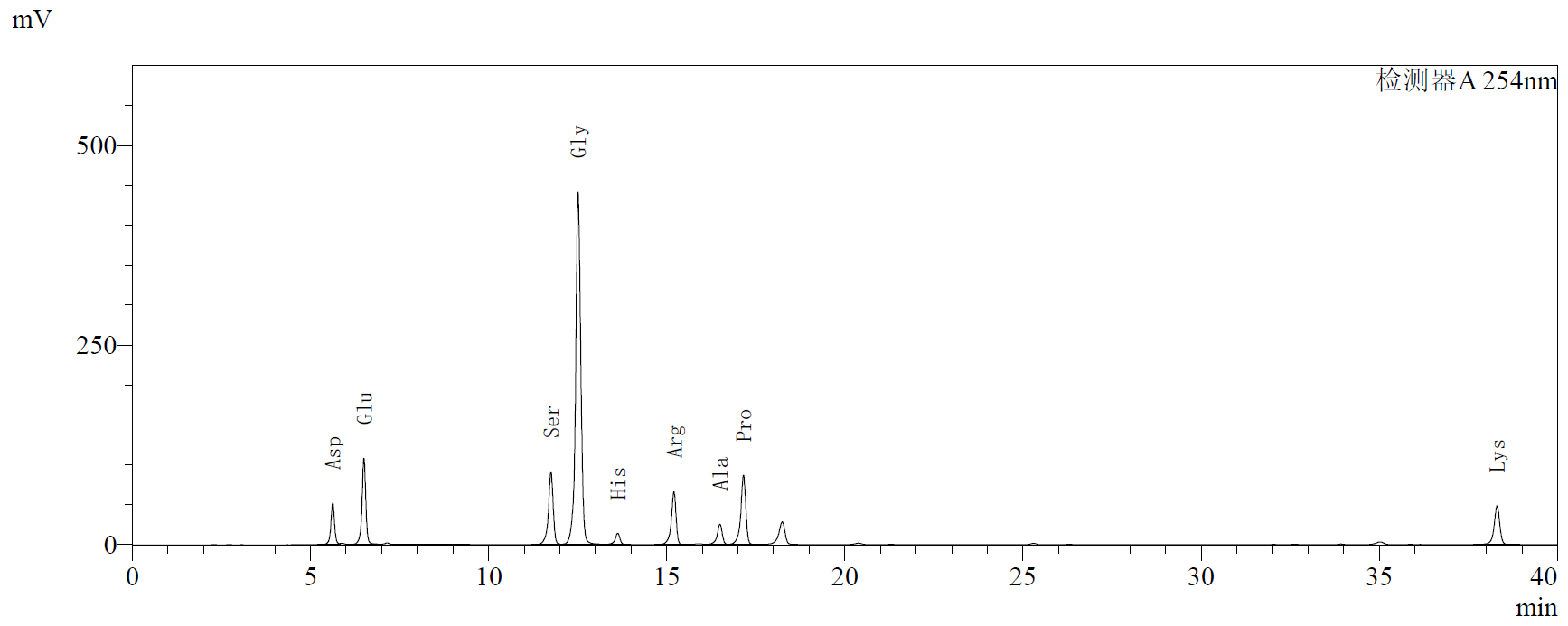


**Fig. S2** Liquid chromatography of hlrGEL6

Supplement: Supplementary file 2 — Additional file 2:Table S1, Fig. S1: Amplification of target gene (gel6) [file 40643_2024_785_MOESM2_ESM.docx]

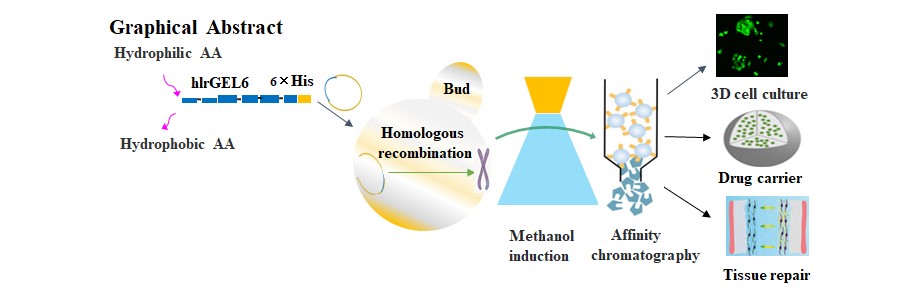

Supplement: Supplementary file 5 — Additional file 5: Additional file 5: Protein sequences of solubility prediction [file 40643_2024_785_MOESM5_ESM.jpg]
