# Supplementary material for: Expression, characterization, and application of human-like recombinant gelatin
Source: Bioresour Bioprocess. 2024 Jul 17;11(1):69. doi: 10.1186/s40643-024-00785-1 (PMC11252100; doi:10.1186/s40643-024-00785-1)
Supplement: Supplementary file 3 — Additional file 3: Additional file3: Fig. S2 Liquid chromatography of hlrGEL6 [file 40643_2024_785_MOESM3_ESM.docx]

**Additional file4** **: Gelation of hlrGEL6**

After being left undisturbed for over 30 minutes, a hydrogel formed at the bottom of the Eppendorf tube containing 200 µL of hlrGEL6 solution (Additional file4: Fig.S3a).

In comparison to commercially available gelatin, the hlrGEL6 hydrogel has a lower minimum gelation concentration of 1.5mg/ml, whereas the minimum concentration for commercially available gelatin is 2.0mg/ml (equivalent to 0.2%) (Additional file4: Fig.S3b).

**
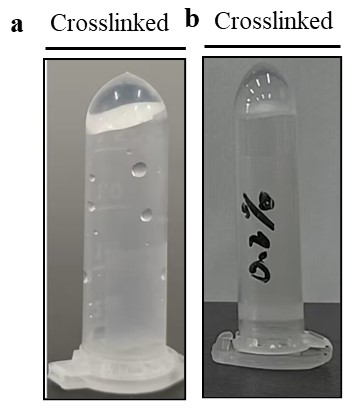
**

**Fig. S3** Gelation of hlrGEL6

**(a)** hlrGEL6 hydrogel formed at the bottom of the Eppendorf tube. **(b)** Control group: commercially available gelatin hydrogel formed at the bottom of the Eppendorf tube
