# Supplementary material for: Expression, characterization, and application of human-like recombinant gelatin
Source: Bioresour Bioprocess. 2024 Jul 17;11(1):69. doi: 10.1186/s40643-024-00785-1 (PMC11252100; doi:10.1186/s40643-024-00785-1)
Supplement: Supplementary file 4 — Additional file 4: Additional file 4: Gelation of hlrGEL6 [file 40643_2024_785_MOESM4_ESM.docx]

**Additional file 5: Protein sequences of solubility prediction**

1. **Online software for predicting protein solubility**

(<https://www.novopro.cn/tools/prot-sol.html>)

**2. Protein sequences are used for prediction**

**(1) Collagen α1 chain III type (COL3A1_Human (Uniprot ID: P02461)**

Mature peptide ：1442AA Solubility: 0.530

QEAVEGGCSHLGQSYADRDVWKPEPCQICVCDSGSVLCDDIICDDQELDCPNPEIPFGECCAVCPQPPTAPTRPPNGQGPQGPKGDPGPPGIPGRNGDPGIPGQPGSPGSPGPPGICESCPTGPQNYSPQYDSYDVKSGVAVGGLAGYPGPAGPPGPPGPPGTSGHPGSPGSPGYQGPPGEPGQAGPSGPPGPPGAIGPSGPAGKDGESGRPGRPGERGLPGPPGIKGPAGIPGFPGMKGHRGFDGRNGEKGETGAPGLKGENGLPGENGAPGPMGPRGAPGERGRPGLPGAAGARGNDGARGSDGQPGPPGPPGTAGFPGSPGAKGEVGPAGSPGSNGAPGQRGEPGPQGHAGAQGPPGPPGINGSPGGKGEMGPAGIPGAPGLMGARGPPGPAGANGAPGLRGGAGEPGKNGAKGEPGPRGERGEAGIPGVPGAKGEDGKDGSPGEPGANGLPGAAGERGAPGFRGPAGPNGIPGEKGPAGERGAPGPAGPRGAAGEPGRDGVPGGPGMRGMPGSPGGPGSDGKPGPPGSQGESGRPGPPGPSGPRGQPGVMGFPGPKGNDGAPGKNGERGGPGGPGPQGPPGKNGETGPQGPPGPTGPGGDKGDTGPPGPQGLQGLPGTGGPPGENGKPGEPGPKGDAGAPGAPGGKGDAGAPGERGPPGLAGAPGLRGGAGPPGPEGGKGAAGPPGPPGAAGTPGLQGMPGERGGLGSPGPKGDKGEPGGPGADGVPGKDGPRGPTGPIGPPGPAGQPGDKGEGGAPGLPGIAGPRGSPGERGETGPPGPAGFPGAPGQNGEPGGKGERGAPGEKGEGGPPGVAGPPGGSGPAGPPGPQGVKGERGSPGGPGAAGFPGARGLPGPPGSNGNPGPPGPSGSPGKDGPPGPAGNTGAPGSPGVSGPKGDAGQPGEKGSPGAQGPPGAPGPLGIAGITGARGLAGPPGMPGPRGSPGPQGVKGESGKPGANGLSGERGPPGPQGLPGLAGTAGEPGRDGNPGSDGLPGRDGSPGGKGDRGENGSPGAPGAPGHPGPPGPVGPAGKSGDRGESGPAGPAGAPGPAGSRGAPGPQGPRGDKGETGERGAAGIKGHRGFPGNPGAPGSPGPAGQQGAIGSPGPAGPRGPVGPSGPPGKDGTSGHPGPIGPPGPRGNRGERGSEGSPGHPGQPGPPGPPGAPGPCCGGVGAAAIAGIGGEKAGGFAPYYGDEPMDFKINTDEIMTSLKSVNGQIESLISPDGSRKNPARNCRDLKFCHPELKSGEYWVDPNQGCKLDAIKVFCNMETGETCISANPLNVPRKHWWTDSSAEKKHVWFGESMDGGFQFSYGNPELPEDVLDVHLAFLRLLSSRASQNITYHCKNSIAYMDQASGNVKKALKLMGSNEGEFKAEGNSKFTYTVLEDGCTKHTGEWSKTVFEYRTRKAVRLPIVDIAPYDIGGPDQEFGVDVGPVCFL

**(2)** **hexamer of unmodified monomer**

peptide ：426AA Solubility: 0.452

GEPGRDGNPGSDGLPGRDGSPGGKGDRGENGSPGAPGAPGHPGPPGGGGSGGGGSGGGGSGGGGSGGGGSGGGGSGEPGRDGNPGSDGLPGRDGSPGGKGDRGENGSPGAPGAPGHPGPPGGGGSGGGGSGGGGSGGGGSGGGGSGGGGSGEPGRDGNPGSDGLPGRDGSPGGKGDRGENGSPGAPGAPGHPGPPGGGGSGGGGSGGGGSGGGGSGGGGSGGGGSGEPGRDGNPGSDGLPGRDGSPGGKGDRGENGSPGAPGAPGHPGPPGGGGSGGGGSGGGGSGGGGSGGGGSGGGGSGEPGRDGNPGSDGLPGRDGSPGGKGDRGENGSPGAPGAPGHPGPPGGGGSGGGGSGGGGSGGGGSGGGGSGGGGS

GEPGRDGNPGSDGLPGRDGSPGGKGDRGENGSPGAPGAPGHPGPPHHHHHH

**(3) hlrGEL6**

peptide ：426AA Solubility: 0.496

GERGDPGSPGNQGQPGNKGSPGPQGPAGQRGNKGERGERGERGASGGGGSGGGGSGGGGSGGGGSGGGGSGGGGSGERGDPGSPGNQGQPGNKGSPGPQGPAGQRGNKGERGERGERGASGGGGSGGGGSGGGGSGGGGSGGGGSGGGGSGERGDPGSPGNQGQPGNKGSPGPQGPAGQRGNKGERGERGERGASGGGGSGGGGSGGGGSGGGGSGGGGSGGGGSGERGDPGSPGNQGQPGNKGSPGPQGPAGQRGNKGERGERGERGASGGGGSGGGGSGGGGSGGGGSGGGGSGGGGSGERGDPGSPGNQGQPGNKGSPGPQGPAGQRGNKGERGERGERGASGGGGSGGGGSGGGGSGGGGSGGGGSGGGGSGERGDPGSPGNQGQPGNKGSPGPQGPAGQRGNKGERGERGERGASHHHHHH
