# Supplementary material for: Expression, characterization, and application of human-like recombinant gelatin
Source: Bioresour Bioprocess. 2024 Jul 17;11(1):69. doi: 10.1186/s40643-024-00785-1 (PMC11252100; doi:10.1186/s40643-024-00785-1)
Supplement: Supplementary file 6 — Additional file 6 [file 40643_2024_785_MOESM6_ESM.docx]

**Additional file 2 Amplification of target gene** **(*gel*6)**

The target gene (*gel*6) was amplified from pUC57-*gel*6 using the *g*el6-F/*gel*6-R primers (Additional file1 2: Table S1) and DNA polymerase. The PCR program consisted of 30 cycles, with each cycle lasting for 40 seconds at 95ºC, followed by 50 seconds at 54ºC and then 120 seconds at 72 ºC. As a result, a 1301bp gene fragment was obtained, which contained restriction enzyme cutting sites, and a 6×His tag, consistent with the predicted results (Fig. S 1).

**Table S1** List of the primers

| **Prime** | **Prime sequence（5^′^→3^′^）** |
| --- | --- |
| *gel*6-F | CTCGAGAAAAGA GGTGAAAGAGGTGATCCAGGTTCTCCAGGTAATCAAGGTCAAC |
| *gel*6-R | GCGGCCGCTTAATGGTGATGGTGATGATGAGAAGCGCCTCTCTCACCACGTTCG |

**
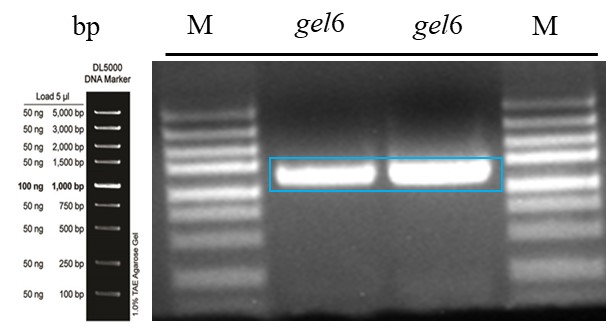
**

**Fig. S1** Gel electrophoresis results for the amplification products of the target gene (*gel*6)
